# Probiotic *Lactobacillus rhamnosus* GG Promotes Mouse Gut Microbiota Diversity and T Cell Differentiation

**DOI:** 10.3389/fmicb.2020.607735

**Published:** 2020-12-17

**Authors:** Chun-wei Shi, Ming-yang Cheng, Xin Yang, Yi-yuan Lu, Hong-duo Yin, Yan Zeng, Ru-yu Wang, Yan-long Jiang, Wen-tao Yang, Jian-zhong Wang, Dan-dan Zhao, Hai-bin Huang, Li-ping Ye, Xin Cao, Gui-lian Yang, Chun-feng Wang

**Affiliations:** ^1^College of Veterinary Medicine, Jilin Agricultural University, Changchun, China; ^2^Jilin Provincial Engineering Research Center of Animal Probiotics, Jilin Agricultural University, Changchun, China; ^3^Key Laboratory of Animal Production and Product Quality Safety of Ministry of Education, Jilin Agricultural University, Changchun, China

**Keywords:** *Lactobacillus rhamnosus*, gut microbiota, T lymphocytes, probiotic, mucosal immunity

## Abstract

Lactic acid bacteria (LAB) are the primary genera of the intestinal flora and have many probiotic functions. In the present study, *Lactobacillus rhamnosus GG* (LGG) ATCC 53103 was used to treat BALB/c mice. After LGG intervention, both low and high LGG doses were shown to improve the observed OTU, Chao1, ACE, and Shannon indices, while the Simpson index decreased, demonstrating that LGG can promote intestinal microbiota abundance and diversity. Furthermore, LGG treatment increased the abundances of intestinal *Firmicutes*, *Bacteroides* and *Actinomycetes* while reducing that of *Proteobacteria*. In addition to its effect on gut the microbiota, LGG could also regulate the host immune system. In the present study, we showed that LGG could affect the percentage of CD3^+^ T lymphocytes in the spleens (SPLs), mesenteric lymph nodes (MLNs), Peyer’s patches (PPs) and lamina propria lymphocytes (LPLs) of mice, including total CD3^+^ T, CD3^+^CD4^+^ T, and CD3^+^CD8^+^ T lymphocytes. Furthermore, LGG could effectively increase the expression of Th1-type cytokines (IFN-γ) and Th2 cytokines (IL-4) in CD4^+^ T cells, indicating that the proportion of Th1 and Th2 cells in mice with LGG treatment was in a high equilibrium state compared to the control group. In addition, the IFN-γ/IL-4 ratio was greater than 1 in mice with LGG intervention, suggesting that LGG tends to mediate the Th1 immune response. The results of the present study also showed that LGG upregulated the expression of IL-17 in CD4^+^ T cells and regulated the percentage of CD4^+^CD25^+^Foxp3^+^ Treg cells in various secondary immunological organs, indicating that LGG may promote the balance of Th-17 and Treg cells.

## Introduction

Lactobacillus is the primary genus of the intestinal flora and probiotics of animals, and it is able to ameliorate the imbalance of the intestinal flora and aid in maintaining a number of biological functions of the immune system (Owyang and Wu, 2014). Studies indicated that *Lactobacillus rhamnosus* LS-8 and *Lactobacillus crustorum* MN047 supplementation possessed the anti-obesity effect on the HFFD fed mice by alleviating inflammatory response and regulating gut microbiota, which further suggested that these two probiotics can be considered as an alternative dietary supplement in combination with the preventive and therapeutic strategies against obesity and related complications (Wang et al., 2020).

Among lactic acid bacteria (LAB), *Lactobacillus rhamnosus* GG (LGG)is the most frequently used strain. LGG strain is a Gram-positive bacteria isolated from the human digestive system for the first time in 1985 by Sherwood Gorbach and Barry Goldin. LGG regulate the intestinal flora through the mechanism of “competitive rejection,” where the colonization of pathogens in the intestinal mucosa is hindered by increasing competition for adhesion sites and nutrients, production of antibacterial compounds (Petrova et al., 2016; Tytgat et al., 2016), regulation of gut microbiota homeostasis (Berni Canani et al., 2016), maintaining function of the intestinal barrier (Zhang et al., 2015), as well as modulation of local or systemic immune response (Johansson et al., 2016). Previous study showed LGG administration enables reprogramming of microbe-microbe interactions and alters ileal microbiota with associated specific CD3^–^CD19^–^cell subset homeostasis. LGG reduces the cell population in the Prevotellaceae NK3B31 group, changes the correlation network in Prevotellaceae NK3B31 group-centric species, and promotes symbiotic synergism of Fusobacterium, Lactobacillus animalis, and Propionibacterium (Zhang et al., 2019). LGG granules administration could decrease the number of *Clostridium perfringens* and increase the *Lactobacillus* and *Bifidobacterium* in the intestine of alcohol-induced mice, which suggested LGG granules prevent alcohol-induced intestinal flora disorder, increase Gram-positive bacteria, decrease Gram-negative bacteria that induce LPS accumulation, and reduce fat accumulation and inflammatory response in liver, so as to ameliorate the liver damage (Gu et al., 2020). LGG supplementation mainly increased the *Bacteroidetes* population in the nude mice. The high abundance of *Bacteroidetes* and *Alistipes* resulted in a high butyrate level in the nude mice treated with LGG to promote butyrate production, protecting against deoxynivalenol exposure in nude mice (Lin et al., 2020).

*Lactobacillus* can regulate both innate and adaptive immunity, playing a role in disease prevention and treatment in the host through immune stimulation and regulation. The effect of *Lactobacillus* on innate immunity is primarily achieved by enhancing the phagocytic ability of the monocyte-phagocytic system. Studies have shown that *Lactobacillus* can enhance the phagocytic activity of mouse peritoneal macrophages and play a regulatory role in the immune system (Donnet-Hughes et al., 1999). In addition, neonatal treatment of p40, a LGG-derived protein, reduced the susceptibility to intestinal injury and colitis and promoted protective immune responses in adult mice, including IgA production and differentiation of regulatory T cells. These findings reveal novel roles of neonatal supplementation of probiotics-derived factors in promoting EGFR-mediated maturation of intestinal functions and innate immunity, which likely promote long-term beneficial outcomes. Furthermore, p40 promotes IgA production through upregulation of APRIL expression in intestinal epithelial cells and mucin production through activation of epidermal growth factor receptor (Shen et al., 2018).

Our recent study also found that LGG can protect the development and integrity of intestinal villi, maintain the integrity of intestinal villi, and promote the growth of villi length. LGG can also regulate the proliferation of T-lymphocytes in the intestine of early weaning piglets at 30 days and 45 days, and increase the number of CD3+ CD4+ T-lymphocytes (Shonyela et al., 2020). Colonization of germ-free mice with either *Lactobacillus johnsonii* (NCC 533) or *Lactobacillus paracasei* (NCC 2461) induced similar germinal center formation and immunoglobulin A-bearing lymphocytes in the mucosa, suggesting that LAB can activate mucosal B-cell responses (Ibnou-Zekri et al., 2003). Our results found that LGG intervention can promote the development and maturation of B lymphocytes, enhance the activation and antigen-presentation ability of B lymphocytes, and regulate the secretion of immunoglobulin by B lymphocytes. Thus, LGG can regulate the mucosal immunity and humoral immunity of mice (Shi et al., 2020).

In addition, the relationship between modulation of gut microbiota and regulation of host immunity by different doses of probiotics is complicated. LGG exerted divergent dose-dependent effects on the intestinal immune cell signaling pathway responses, with 9-doses LGG being more effective in activating the innate TLR9 signaling pathway than 14-doses in the HGM pigs vaccinated with AttHRV (Wang et al., 2020). Thus, in the present study, by using different doses of LGG to intervene in mice, we found that the changes of intestinal flora caused by different doses of lactic acid bacteria had different effects on the proliferation and differentiation of T lymphocyte subsets in mice. The application measurement of lactic acid bacteria is still to be discussed.

## Results

### Alpha Diversity of the Gut Microbiota

After quality trimming and chimera checking, 576,311 high-quality 16S rRNA gene reads were obtained from the 15 samples that had an average length of 437.10 bp. The Good’s coverage index was greater than 99%, and 6390 OTUs were identified from all samples. Rarefaction, Shannon, Coverage and OTU rank-abundance curve analysis were used to standardize and compare the observed taxon richness among samples and to determine whether the contents of all samples were unequally sampled. The results suggested that the sequencing depth was sufficient to cover the microbial diversity of each sample and that additional data would not yield more OTUs (Supplementary Figure 1).

**FIGURE 1:**
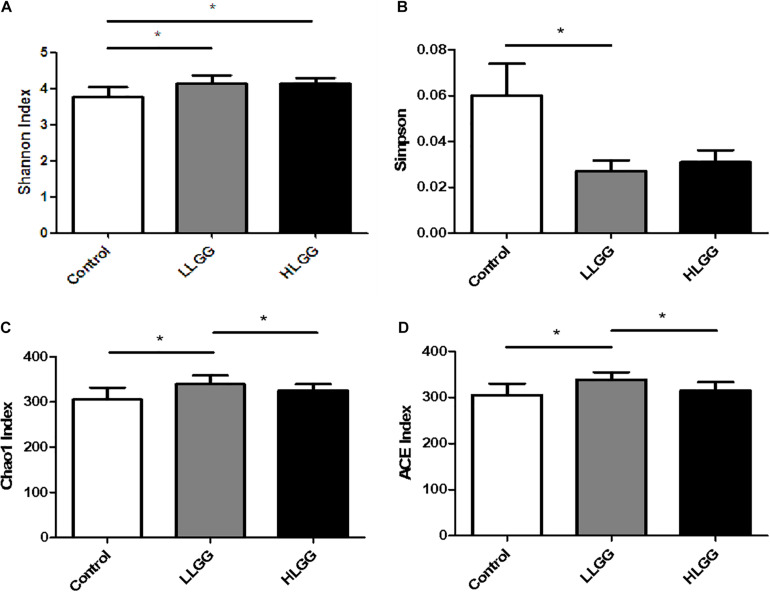
Analysis of different microbial alpha diversity indices in the three experimental groups. The Shannon **(A)** and Sipson **(B)** indices were used as diversity estimators. The Chao1 **(C)** and ACE **(D)** indices were used as richness estimators. *Indicated significant differences (*p* < 0.05) by students’ *t*-test.

In the present study, the Chao1, ACE, Simpson, and Shannon indices were used to evaluate the richness and alpha diversity of the gut microbiota (Figure 1). The Chao1 and Ace indices can be used to evaluate the richness of the intestinal microbiota, which is proportional to the richness of the gut microbiota, while the Simpson and Shannon indices can be used to evaluate the diversity of the gut microbiota, higher values of which indicate lower and greater microbiota diversity, respectively. The results showed that after 7 LGG interventions, compared to the control group, the Chao1, ACE, and Shannon indices in the LLGG group significantly increased, whereas the Simpson index significantly decreased, indicating that the low-dose LGG treatment could promote the richness and diversity of the gut microbiota. Compared to the control group, the Shannon index significantly increased in the HLGG group, while no significant differences were observed between the Chao1, ACE, and Simpson indices, indicating that the high-dose LGG treatment could promote gut microbiota diversity. Compared to the HLGG group, the Chao1 and ACE indices in the LLGG group significantly increased, while the Shannon and Simpson indices showed no significant changes. Thus, based on the observed Chao1, ACE, Simpson, and Shannon indices, LGG could promote the alpha diversity of gut microbiota, and the low-dose LGG treatment could better promote the richness and diversity of the gut microflora.

### LGG Pretreatment Affects the Gut Microbiota Structure

**FIGURE 2:**
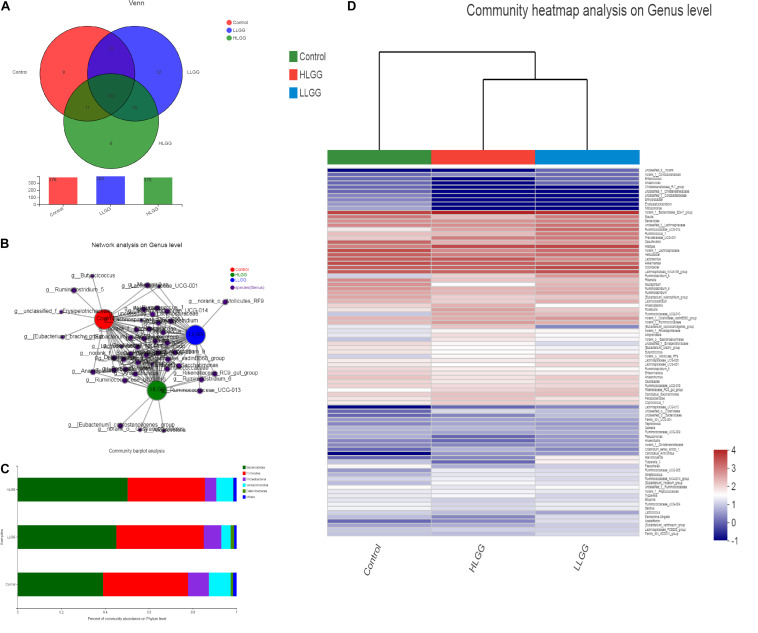
Changes in gut microbiota communities of BALB/c mice in response to LGG pretreatment. **(A)** Venn diagram of common and unique OTUs among the three groups. The numbers of observed OTUs sharing ≥97% nucleotide sequence identity. **(B)** A network diagram showing the OTUs among the three groups. **(C)** Gut microbiota communities at the phylum level. The stacked bars show the combined relative abundances of phylum-level taxa per animal. Colors are assigned for all phyla detected. **(D)** Heatmap showing the spatial distributions of all OTUs at the genus level.

Venn diagrams were generated to evaluate the distribution of OTUs among the different treatment groups. As shown in Figure 2A, the taxon-independent Venn analysis results showed that 333 OTUs were shared among the three groups, with the total number of OTUs being highest in the HLGG group, followed by the LLGG and control groups. The unique number of OTUs was 9, 12, and 6 in the control, LLGG and HLGG groups, respectively. The predominant genera co-occurred among the three groups, while the abundances of some rare genera differed. The genera *Butyricicoccus*, Ruminiclostridium_5, *[Eubacterium]_brachy_group* and *unclassified_f__Erysipelotrichaceae* were unique to the control group. The genera *norank_o__Mollicutes_RF9* were unique to the LLGG group. The genera *norank_o__Gastranaerophilales*, *Alloprevotella* and *[Eubacterium]_coprostanoligenes_group* were unique to the HLGG group (Figure 2B) To elucidate the effect of LGG on the composition and structure of the gut microflora, we assessed the community composition of each sample at the phylum and genus levels. The sequencing results showed the presence of 10 phyla, of which *Firmicutes* and *Bacteroidetes* were the primary phyla in all three treatment groups. In addition, *Verrucomicrobia*, *Deferribacteres*, and *Proteobacteria* were present in all samples. In the control group, the relative abundances of *Bacteroidetes*, *Firmicutes*, *Verrucomicrobia*, *Proteobacteria*, and *Deferribacteres* were 39.00, 38.85, 9.51, 9.91, and 1.16%, respectively. In the LLGG group, the relative abundances of *Bacteroidetes* and *Firmicutes* increased to 45.12 and 39.89%, while those of *Verrucomicrobia*, *Proteobacteria*, and *Deferribacteres* decreased to 8.05, 4.24, and 1.62%, respectively. In the HLGG group, the relative abundance of *Bacteroidetes* increased to 50.26%, while the abundances of *Firmicutes*, *Verrucomicrobia* and *Proteobacteria* and *Deferribacteres* decreased to 35.30, 5.23, 7.64, and 0.16%, respectively (Figure 2C). The heatmap-based analysis at genus level showed that the gut microbiota profiles of mice in the LLGG group were similar to those in the HLGG group (Figure 2D).

**FIGURE 3:**
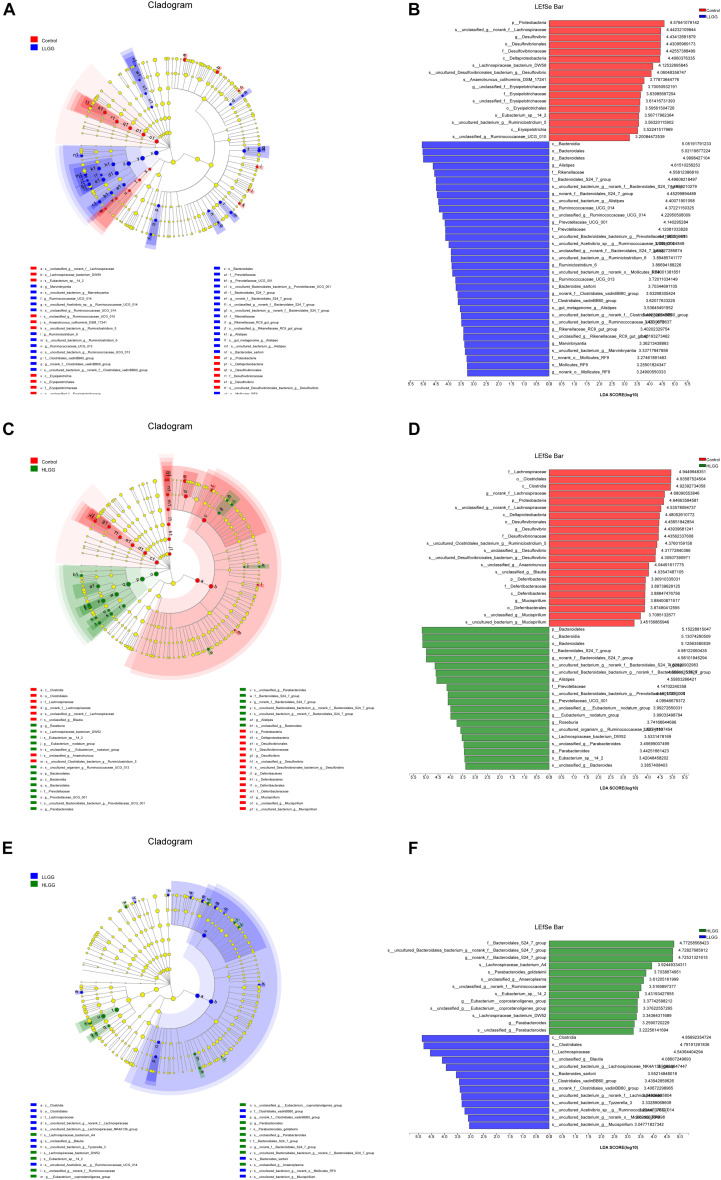
LEfSe analysis results. Only the taxa with LDA scores higher than 3 are shown; **(A,B)** Cladogram showing the phylogenetic relationships of bacterial taxa and LDA scores between the control and LLGG groups. **(C,D)** Cladogram showing the phylogenetic relationships of bacterial taxa and LDA scores between the control and HLGG groups. **(E,F)** Cladogram showing the phylogenetic relationships of bacterial taxa and LDA scores between the LLGG and HLGG groups.

**FIGURE 4:**
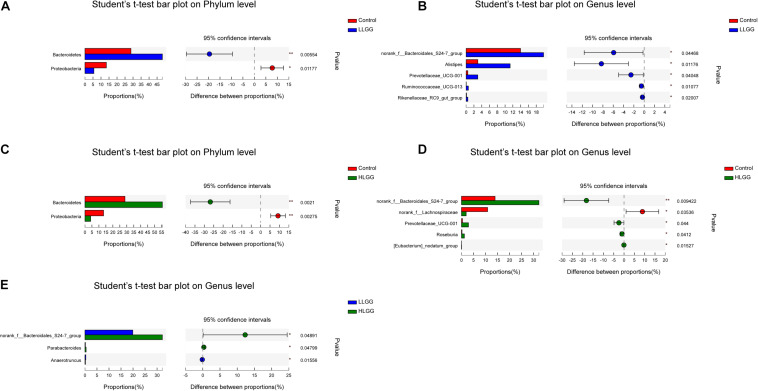
Differences in the relative abundances of OTUs from the three indicated treatment groups were analyzed using Student’s *t*-test. **(A–E)** The taxonomy of the OTUs (phylum and genus levels) is depicted on the left. The *Y*-axis shows the species name at a specific taxonomic level, and the *X*-axis shows the average relative abundance in different groups. The columns with different colors represent different groups, and the rightmost value is the *P*-value, *0.01 < *P* ≤ 0.05, **0.001 < *P* ≤ 0.01.

The PLS-DA results based on the relative abundances of OTUs clearly showed distinguishable ileal mucosal microbiota samples among the three groups (Supplementary Figure 2). To identify the specific phylotypes that were significantly altered in response to LGG supplementation, all effective sequences from the sample were analyzed using the LEfSe method When comparing the control and LLGG groups (Figures 3A,B), the relative abundance of *Proteobacteria* was enriched at the phylum level, while the relative abundances of *unclassified_f__Erysipelotrichaceae* and *Desulfovibrio* were enriched at the genus level in the control group. In contrast, in the LLGG group, the relative abundance of *Bacteroides* was enriched at the phylum level, and the relative abundances of *Marvinbryantia*, *Ruminococcaceae_UCG_014*, *Ruminiclostridium_6*, *Ruminococcaceae_UCG_013*, *norank_f__ Clostridiales_vadinBB60_group*, *Prevotellaceae_UCG_001*, *nor ank_f__Bacteroidales_S24_7_group*, *Rikenellaceae_RC9_gut_ group*, *Alistipes*, and *norank_o__Mollicutes_RF9* were enriched at the genus level. When comparing the control and HLGG groups (Figures 3C,D), the relative abundance of *Bacteroidetes* was enriched at the phylum level, while the relative abundances of *Roseburia*, *Eubacterium__nodatum_group*, *Prevotellaceae_UCG_001*, *Parabacteroides*, *norank_f__Bacteroidales_S24_7_group*, and *Alistipes* were enriched at the genus level in the HLGG group. In contrast, in the control group, the relative abundances of *Proteobacteria* and *Deferribacteres* were enriched at the phylum level, while those of *norank_f__Lachnospiraceae*, *Desulfovibrio*, and *Mucispirillum* were enriched at the genus level. In addition, when comparing the LLGG and HLGG groups (Figures 3E,F), the relative abundance of *norank_f__Clostridiales_vadinBB60_group* was enriched at the genus level in the LLGG group, while the relative abundances of *Eubacterium__coprostanoligenes_group* and *norank_f__Bacteroidales_S24_7_group* were enriched in the LHGG group. Subsequently, Student’s *t*-test was used to evaluate the LEfSe results. Compared to the control group, the relative abundance of *Bacteroides* significantly increased in the LLGG group, whereas that of *Proteobacteria* significantly decreased at the phylum level (Figure 4A), and the relative abundances of *norank_f__Bacteroidales_S24-7_group*, *Alistipes*, *Prevotellaceae_UCG-001*, *Ruminococcaceae_UCG-013*, and *Rikenellaceae_RC9_gut_group* significantly increased at the genus level (Figure 4B). Compared to the control group, the relative abundance of *Bacteroides* significantly increased in the HLGG group, while that of *Proteobacteria* significantly decreased at the phylum level (Figure 4C), and at the genus level, the relative abundances of *norank_f__Bacteroidales_S24-7_group*, *Prevotellaceae_UCG-001*, *Roseburia*, and *[Eubacterium]_nodatum_group* significantly increased, and that of *norank_f__Lachnospiraceae* significantly decreased (Figure 4D). Compared to the LLGG group, no significant difference was observed in the HLGG group at the phylum level, whereas at the genus level, the relative abundances of *norank_f__Bacteroidales_S24-7_group* and *Parabacteroides* were significantly increased and that of *Anaerotruncus* was significantly decreased (Figure 4E).

In addition, to characterize the functional changes of intestinal microbiome under LGG intervention, we predicted the functional composition profiles from 16S rRNA sequencing data with PICRUSt in HLGG, LLGG, and controls. Based on three groups of mice intestinal flora KEGG pathway (level 3) abundance of analysis, we found enrichment of intestinal flora pathways in LLGG group, including Biosynthesis of amino acids, Carbon metabolism, ABC transporters, and Ribosome pathways, etc (Supplementary Figure 3).

### Effect of LGG on the Proportion of T Cells

**FIGURE 5:**
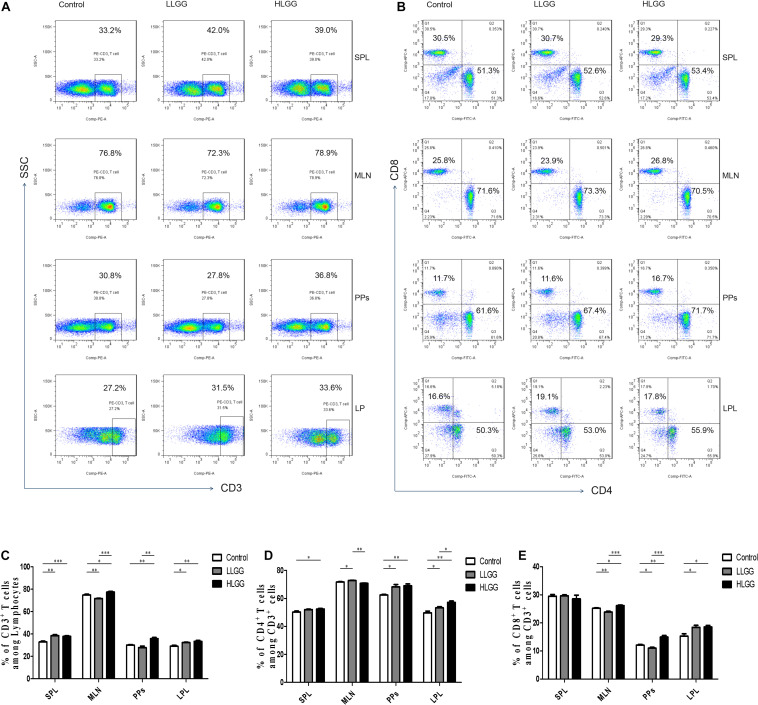
LGG treatment increased the percentage of CD3^+^, CD3^+^CD4^+^, and CD3^+^CD8^+^ T cells in various secondary immunological organs. **(A)** Changes in the proportion of CD3^+^ T cells in the SPLs, MLNs, PPs, and LPLs of mice treated with different doses of LGG, as shown by representative flow cytometry data. **(B)** Changes in the proportion of CD3^+^CD4^+^ T cells and CD3^+^CD8^+^ T cells in the SPLs, MLNs, PPs, and LPLs of mice treated with different LGG doses, as shown by representative flow cytometry data. **(C)** Statistical data showing the percentage of CD3^+^ T cells in various secondary immunological organs from **(A)**. **(D,E)** Statistical data showing the percentage of CD3^+^CD4^+^ T cells and CD3^+^CD8^+^ T cells in various secondary immunological organs from **(B)**. *0.01 < *P* ≤ 0.05, **0.001 < *P* ≤ 0.01, ***P ≤ 0.001.

CD3 participates in signal transduction after antigen recognition by TCR, and all T cells express CD3 molecules. After 7 LGG interventions, the percentages of CD3^+^ T cells in the spleens (SPLs) of mice in the control, LLGG and HLGG groups were 32.9 ± 0.68%, 38.52 ± 0.88%, and 37.86 ± 0.88%, respectively. Compared to the percentage of CD3^+^ T cells in the SPLs of mice in the control group, a higher percentage was observed in those of LLGG group (*p* < 0.05, *p* = 0.001) and HLGG group (*p* < 0.05, *p* = 0.0007). The percentages of CD3^+^ T cells in the mesenteric lymph nodes (MLNs) of mice in the control, LLGG and HLGG groups were 74.84 ± 0.71%, 71.72 ± 0.27%, and 77.48 ± 0.63%, respectively. The percentage of CD3^+^ T cells in the MLNs was lower in the LLGG group (*p* < 0.05, *p* = 0.0035) and higher in the HLGG group (*p* < 0.05, *p* = 0.0246) than that observed in the control group. The percentages of CD3^+^ T cells in lamina propria lymphocytes (LPLs) in the control, LLGG and HLGG groups were 29.04 ± 0.89%, 32.30 ± 0.38%, and 33.38 ± 0.82%, respectively. Compared to the percentage of CD3^+^ T cells observed in the LPLs of mice from the control group, higher percentages were observed in the LLGG group (*p* < 0.05, *p* = 0.0101) and HLGG group (*p* < 0.05, *p* = 0.0072). The percentages of CD3^+^ T cells in Peyer’s patches (PPs) of mice in the control, LLGG and HLGG groups were 30.04 ± 0.50%, 27.82 ± 1.42%, and 35.86 ± 1.41%, respectively. The percentage of CD3^+^ T cells in PPs from mice in the HLGG group was higher than that observed in the control group (*p* < 0.05, *p* = 0.001) and LLGG group (*p* < 0.05, *p* = 0.0018) (Figures 5A,C).

CD4 only exists in T cells that can recognize exogenous antigens presented by MHC-II, and CD3^+^CD4^+^ T cells are important immune cells. After 7 LGG interventions, the percentages of CD3^+^ CD4^+^ T cells among CD3^+^ T lymphocytes in the SPL in the control and HLGG groups were 50.30 ± 0.72% and 52.48 ± 0.41%, respectively. The percentage of CD3^+^ CD4^+^ T cells among CD3^+^ T lymphocytes in the SPLs of mice in the HLGG group was higher than that observed in the control group (*p* < 0.05, *p* = 0.0310). The percentages of CD3^+^ CD4^+^ T cells among CD3^+^ T lymphocytes in MLNs from mice in the control, LLGG and HLGG groups were 71.76 ± 0.24%, 72.62 ± 0.27% and 70.96 ± 0.25%, respectively. The percentages of CD3^+^ CD4^+^ T cells among CD3^+^ T lymphocytes in PPs from the control, LLGG and HLGG groups were 62.48 ± 0.46%, 68.30 ± 1.60% and 69.08 ± 1.29%, respectively. Compared to the percentage of CD3^+^ CD4^+^ T cells observed among CD3^+^ T lymphocytes in PPs in the control group, higher percentages were observed in the LLGG group (*p* < 0.05, *p* = 0.0101) and HLGG group (*p* < 0.05, *p* = 0.0014). The percentages of CD3^+^ CD4^+^ T cells among CD3^+^ T lymphocytes in the LPLs from the control, LLGG and HLGG groups were 49.76 ± 1.23%, 53.30 ± 0.84% and 57.20 ± 0.94%, respectively. The percentage of CD3^+^ CD4^+^ T cells among CD3^+^ T lymphocytes in the LPLs from the HLGG group was higher than that observed in the control group (*p* < 0.05, *p* = 0.0014) and LLGG group (*p* < 0.05, *p* = 0.0018), while the percentage observed in the LLGG group was also higher than that measured in the control group (*p* < 0.05, *p* = 0.0452) (Figures 5B,D).

CD8 exists only in T cells that can recognize endogenous antigens presented by MHC-I and can kill abnormal cells, namely, cytotoxic T cells. After 7 LGG interventions, the percentages of CD3^+^ CD8^+^ T cells among CD3^+^ T lymphocytes in SPLs from the control, LLGG and HLGG groups was not significantly different. The percentages of CD3^+^ CD8^+^ T cells among CD3^+^ T lymphocytes in the MLNs from the control, LLGG and HLGG groups were 25.22 ± 0.17%, 23.86 ± 0.28%, and 26.16 ± 0.23%, respectively. The percentage of CD3^+^ CD8^+^ T cells among CD3^+^ T lymphocytes in MLNs from the HLGG group was higher than that observed in the control group (*p* < 0.05, *p* = 0.0129) and LLGG group (*p* < 0.05, *p* = 0.0003), while the percentage in the LLGG group was lower than that observed in the control group (*p* < 0.05, *p* = 0.0037). The percentages of CD3^+^ CD8^+^ T cells among CD3^+^ T lymphocytes in PPs from the control, LLGG and HLGG groups were 12.08 ± 0.26%, 11.01 ± 0.30%, and 14.96 ± 0.57%, respectively. The percentage of CD3^+^ CD8^+^ T cells among CD3^+^ T lymphocytes in PPs from the HLGG group was higher than that observed in the control group (*p* < 0.05, *p* = 0.0018) and LLGG group (*p* < 0.05, *p* = 0.0003), while the percentage in the LLGG group was lower than that observed in the control group (*p* < 0.05, *p* = 0.0291). The percentages of CD3^+^ CD8^+^ T cells among CD3^+^ T lymphocytes in the LPLs from the control, LLGG and HLGG groups were 15.28 ± 0.83%, 18.42 ± 0.73%, and 18.56 ± 0.52%, respectively. Compared to the percentage of CD3^+^ CD8^+^ T cells observed among CD3^+^ T lymphocytes in the LPLs from the control group, a higher percentage was observed in the LLGG group (*p* < 0.05, *p* = 0.0225) and HLGG group (*p* < 0.05, *p* = 0.0105) (Figures 5B,E).

In summary, both low-dose and high-dose LGG intervention significantly affected the percentage of CD3^+^ T, CD3^+^ CD4^+^ T and CD3^+^ CD8^+^ T cells in peripheral lymphoid organs (SPLs), intestinal secondary lymphoid organs (MLNs, PPs) and intestinal tissue (LP). In the LLGG group, a significant increase the percentage of CD3^+^ T cells in the SPLs, CD3^+^ CD4^+^ T cells in the MLNs and PPs, and CD3^+^, CD3^+^CD4^+^, and CD3^+^CD8^+^ T cells in the LPLs was observed, while a significant decrease in the percentage of CD3^+^ T and CD3^+^ CD8^+^ T cells was observed in the MLNs and PPs. Thus, a high dose of LGG had a strong effect on the percentage of all subsets of T cells, which can better improve the immune function of mice.

### Effects of LGG on Th1 Cell Differentiation in Mice

BALB/c mice at 6 weeks of age received 7 high- or low-dose LGG treatments on alternate days, and a PBS control group was also established. SPLs, MLNs, PPs, and LP of the three groups of mice were isolated after the interventions to prepare single-cell suspensions. PMA, ionomycin, and BFA were added to the single-cell suspensions. Lymphocytes were collected 6 h later, and IFN-γ was labeled with a specific fluorescent antibody and detected by flow cytometry. IFN-γ, also known as type II interferon, is one of the most important types of Th1 cytokines.

**FIGURE 6:**
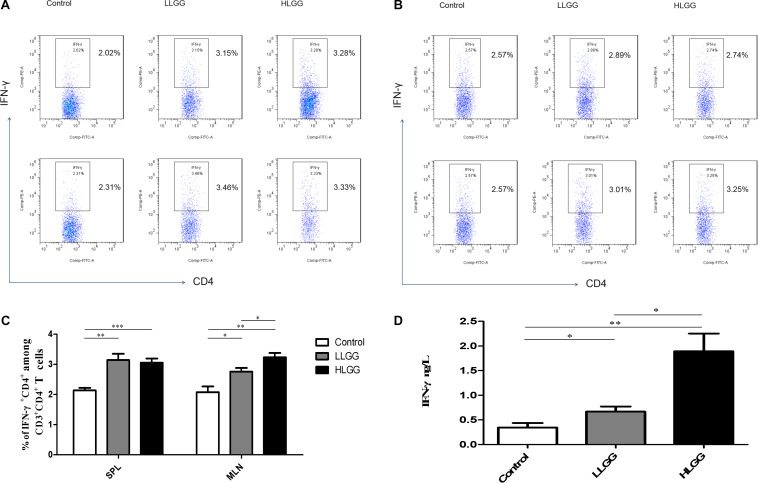
LGG treatment increased the percentage of CD3^+^CD4^+^ IFN-γ^+^ Th1 cells in secondary immunological organs and the serum IFN-γ concentration. **(A)** Changes in the proportion of CD3^+^CD4^+^IFN-γ^+^ Th1 cells in the SPLs shown by representative flow cytometry data. **(B)** Changes in the proportion of CD3^+^CD4^+^IFN-γ^+^ Th1 cells in the MLNs shown by representative flow cytometry data. **(C)** Statistical data showing the percentage of CD3^+^CD4^+^ IFN-γ^+^ Th1 cells in the SPLs and MLNs from **(A,B)**. **(D)** Serum IFN-γ levels in mice treated with LGG as detected by ELISA. *0.01 < *P* ≤ 0.05, **0.001 < *P* ≤ 0.01, ***P ≤ 0.001.

After 7 LGG interventions, the percentages of CD3^+^CD4^+^ IFN-γ^+^ Th1 cells in the SPLs in the control, LLGG and HLGG groups were 2.14 ± 0.08%, 3.142 ± 0.20%, and 3.054 ± 0.14%, respectively. Compared to the percentage of CD3^+^CD4^+^ IFN-γ^+^ Th1 cells in the SPLs from the control group, a higher percentage was observed in the LLGG group (*p* < 0.05, *p* = 0.0020) and HLGG group (*p* < 0.05, *p* = 0.0005) (Figures 6A,C). The percentages of CD3^+^CD4^+^ IFN-γ^+^ Th1 cells in the MLNs from the control, LLGG and HLGG groups were 2.080 ± 0.19%, 2.760 ± 0.12%, and 3.236 ± 0.14%, respectively. Compared to the percentage of CD3^+^CD4^+^ IFN-γ^+^ Th1 cells in the MLNs from the control group, a higher percentage was observed in the LLGG group (*p* < 0.05, *p* = 0.0166) and HLGG group (*p* < 0.05, *p* = 0.0012). The percentage of CD3^+^CD4^+^ IFN-γ^+^ Th1 cells in the MLNs from the HLGG group was also higher than that observed in the LLGG group (*p* < 0.05, *p* = 0.0351) (Figures 6B,C). Compared to the control group, the LLGG and HLGG groups both exhibited increased percentages of Th1 cells. In addition, the percentage of Th1 cells in the MLNs from the HLGG group was higher than that observed in the LLGG group.

After 7 LGG interventions, serum IFN-γ concentrations were detected by ELISA. Compared to the concentration of serum IFN-γ observed in the control group, a higher concentration was observed in the LLGG group (*p* < 0.05, *p* = 0.0091) and HLGG group (*p* < 0.05, *p* = 0.0021). The concentration of serum IFN-γ in the HLGG group was also higher than that observed in the LLGG group (*p* < 0.05, *p* = 0.0073) (Figure 6D).

### Effects of LGG on Th2 Cell Differentiation in Mice

**FIGURE 7:**
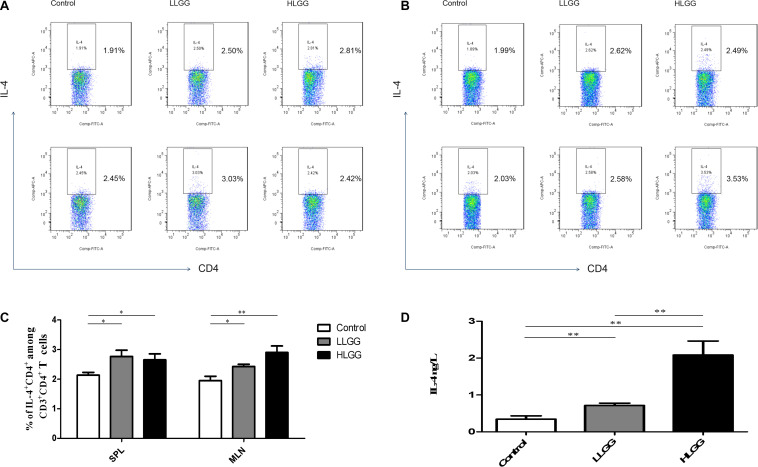
LGG treatment increased the percentages of CD3^+^CD4^+^IL-4^+^ Th2 cells in secondary immunological organs and the serum IL-4 concentration. **(A)** Changes in the proportion of CD3^+^CD4^+^IL-4^+^ Th2 cells in the SPLs shown by representative flow cytometry data. **(B)** Changes in the proportion of CD3^+^CD4^+^IL-4^+^ Th2 cells in the MLNs shown by representative flow cytometry data. **(C)** Statistical data showing the percentage of CD3^+^CD4^+^ IL-4^+^ Th2 cells in the SPLs and MLNs from **(A,B)**. **(D)** Serum IL-4 levels in mice treated with LGG as detected by ELISA. *0.01 < *P* ≤ 0.05, **0.001 < *P* ≤ 0.01.

IL-4 molecules are primarily secreted by Th2 cells and can promote B cells to proliferate and induce CD4^+^ T cells to differentiate into Th2 cells. After 7 LGG interventions, the percentages of CD3^+^CD4^+^IL-4^+^ Th2 cells in the SPL in the control, LLGG and HLGG groups were 2.142 ± 0.0955%, 2.730 ± 0.218%, and 2.660 ± 0.14%, respectively. Compared to the percentage of CD3^+^CD4^+^IL-4^+^ Th2 cells observed in the SPLs from the control group, a higher percentage was observed in the LLGG group (*p* < 0.05, *p* = 0.0384) and HLGG group (*p* < 0.05, *p* = 0.0155) (Figures 7A,C). The percentages of CD3^+^CD4^+^IL-4^+^ Th2 cells in the MLNs from the control, LLGG and HLGG groups were 1.952 ± 0.147%, 2.428 ± 0.077%, and 2.900 ± 0.223%, respectively. Compared to the percentage of CD3^+^CD4^+^IL-4^+^ Th2 cells in the MLNs from the control group, a higher percentage was observed in the LLGG group (*p* < 0.05, *p* = 0.0212) and HLGG group (*p* < 0.05, *p* = 0.0076) (Figures 7B,C). Compared to the control group, the percentage of Th2 cells was decreased in both the LLGG and HLGG groups.

After 7 LGG interventions, the serum IL-4 concentration was detected by ELISA. Compared to the concentration of serum IL-4 observed in the control group, a higher concentration was observed in the LLGG group (*p* < 0.05, *p* = 0.0472) and HLGG group (*p* < 0.05, *p* = 0.0032). The concentration of serum IL-4 in the HLGG group was also higher than that observed in the LLGG group (*p* < 0.05, *p* = 0.0117) (Figure 7D).

IFN-γ can promote the differentiation of Th0 cells into Th1 cells and affect the ratio of Th1/Th2, while IL-4 can promote the differentiation of Th0 cells into Th2 cells. Th1 cells primarily mediate immune responses related to cellular and local inflammation and participate in cellular immunity. The primary function of Th2 cells is to stimulate B cell proliferation and produce antibodies, which are involved in humoral immunity. After LGG intervention, the ratio of IFN-γ/IL-4 expressed by CD4^+^ T cells in the SPLs and MLNs was greater than 1 (Supplementary Figures 4A,B). The ratio of IFN-γ/IL-4 in serum detected by ELISA was also greater than 1 (Supplementary Figure 4C). These results suggest that LGG tends to mediate the Th2 immune response.

### Effects of LGG on Th17 Cell Differentiation in Mice

**FIGURE 8:**
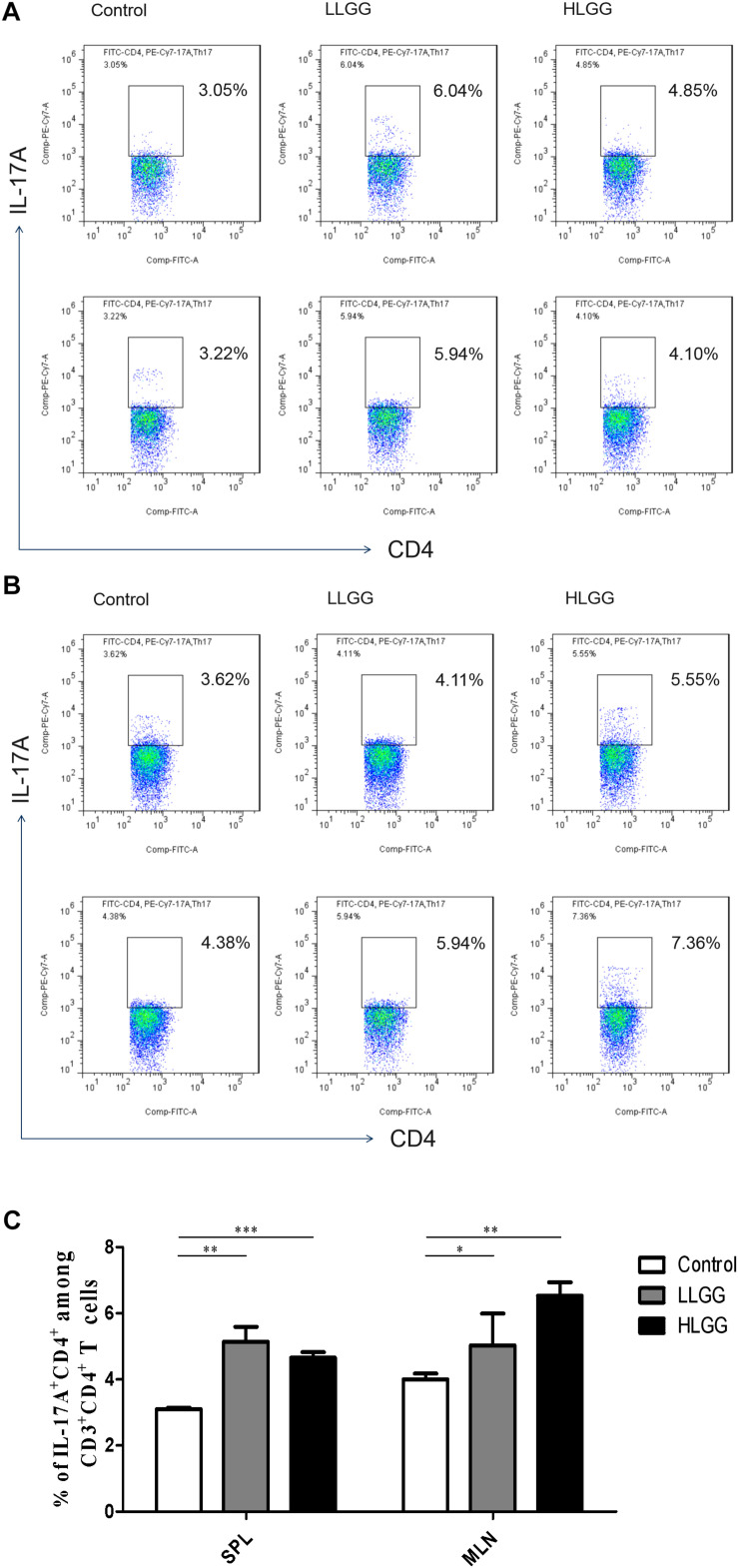
LGG treatment increased the percentage of CD3^+^CD4^+^IL-17^+^ Th17 cells in secondary immunological organs. **(A)** Changes in the proportion of CD3^+^CD4^+^IL-17^+^ Th17 cells in the SPLs shown by representative flow cytometry data. **(B)** Changes in the proportion of CD3^+^CD4^+^IL-17^+^ Th17 cells in the MLNs shown by representative flow cytometry data. **(C)** Statistical data showing the percentage of CD3^+^CD4^+^IL-17^+^ Th17 cells in the SPL and MLN from **(A,B)**. *0.01 < *P* ≤ 0.05, **0.001 < *P* ≤ 0.01, ***P ≤ 0.001.

Th17 cells represent a small proportion of CD4^+^ T cells that express IL-17. After 7 LGG interventions, the percentages of CD3^+^CD4^+^ IL-17^+^ Th17 cells in the SPLs from the control, LLGG and HLGG groups were 3.104 ± 0.038%, 5.144 ± 0.44%, and 4.656 ± 0.16%, respectively. Compared to the percentage of CD3^+^CD4^+^IL-17^+^ Th17 cells in the SPLs from the control group, a higher percentage was observed in the LLGG group (*p* < 0.05, *p* = 0.0018) and HLGG group (*p* < 0.05, *p* = 0.0001) (Figures 8A,C). The percentages of CD3^+^CD4^+^IL-17^+^ Th17 cells in the MLNs from the control, LLGG and HLGG groups were 4.003 ± 0.17%, 5.390 ± 0.42%, and 6.528 ± 0.40%, respectively. Compared to the percentage of CD3^+^CD4^+^IL-17^+^ Th17 cells from the MLNs in the control group, a higher percentage was observed in the LLGG group (*p* < 0.05, *p* = 0.0242) and HLGG group (*p* < 0.05, *p* = 0.0012) (Figures 8B,C). No significant difference was observed in the percentage of CD3^+^CD4^+^IL-17^+^ Th17 cells between the LLGG and HLGG groups. Compared to the control group, both the LLGG and HLGG groups showed an increase in the percentage of Th17 cells.

### Effect of LGG on CD4+CD25+Foxp3+ Treg Subsets in Mice

**FIGURE 9:**
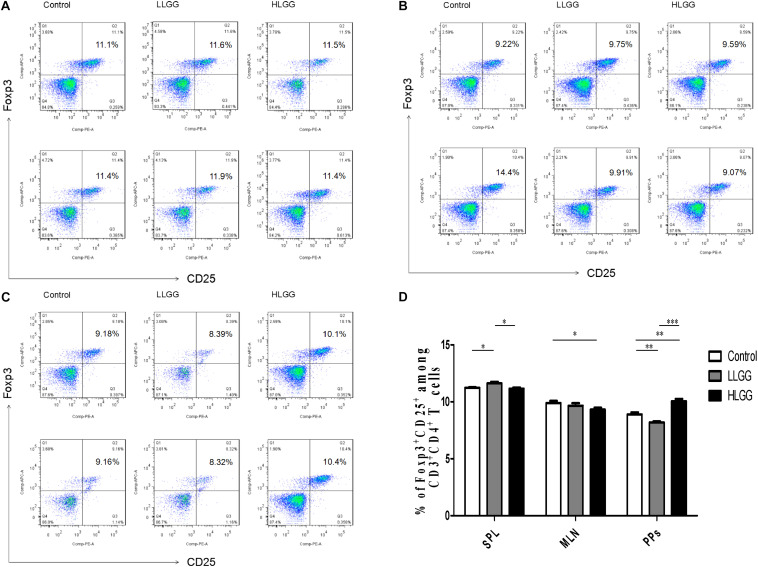
LGG treatment affected the percentage of CD4^+^CD25^+^Foxp3^+^ Treg cells in secondary immunological organs. **(A)** Changes in the proportion of CD4^+^CD25^+^Foxp3^+^ Treg cells in the SPLs shown by representative flow cytometry data. **(B)** Changes in the proportion of CD4^+^CD25^+^Foxp3^+^ Treg cells in the MLN shown by representative flow cytometry data. **(C)** Changes in the proportion of CD4^+^CD25^+^Foxp3^+^ Treg cells in PPs shown by representative flow cytometry data. **(D)** Statistical data showing the percentage of CD4^+^CD25^+^Foxp3^+^ Treg cells in the SPLs, MLNs, and PPs from **(A–C)**. *0.01 < *P* ≤ 0.05, **0.001 < *P* ≤ 0.01, ***P ≤ 0.001.

Treg cells express CD4 and CD25, as well as the transcription factor Foxp3, which controls their development. After 7 LGG interventions, the percentages of CD4^+^CD25^+^Foxp3^+^ Treg cells in the SPLs from the control, LLGG and HLGG groups were 11.23 ± 0.05%, 11.62 ± 0.13%, and 11.16 ± 0.09%, respectively. The mice in the LLGG group had a higher percentage of CD4^+^CD25^+^Foxp3^+^ Treg cells in the SPL than those in the control group (*p* < 0.05, *p* = 0.0311) and HLGG group (*p* < 0.05, *p* = 0.0251) (Figures 9A,D). The percentages of CD4^+^CD25^+^Foxp3^+^ Treg cells in the MLNs from the control and HLGG groups were 9.896 ± 0.1913% and 9.314 ± 0.16%, respectively. Compared to the percentage of CD4^+^CD25^+^Foxp3^+^ Treg cells observed in the MLNs from the control group, a lower percentage was observed in the MLNs from the HLGG group (*p* < 0.05, *p* = 0.0492) (Figures 9B,D). The percentages of CD4^+^CD25^+^Foxp3^+^ Treg cells in PPs from the control, LLGG and HLGG groups were 8.898 ± 0.17%, 8.194 ± 0.09%, and 10.05 ± 0.20%, respectively. Compared to the percentage of CD4^+^CD25^+^Foxp3^+^ Treg cells in PPs from the control group, a lower percentage was observed in the LLGG group (*p* < 0.05, *p* = 0.0069), while a higher percentage was observed in the HLGG group (*p* < 0.05, *p* = 0.0028). The percentage of CD4^+^CD25^+^Foxp3^+^ Treg cells in PPs from the HLGG group was significantly higher than that observed in the LLGG group (*p* < 0.05, *p* < 0.0001) (Figures 9C,D). Compared to the control group, the LLGG group exhibited an increased percentage of Treg cells in the SPLs and a decreased percentage of Treg cells in the MLNs and PPs, while the HLGG group had a decreased percentage of Treg cells in the MLNs and an increased percentage of Treg cells in the PPs.

In summary, LGG could affect the differentiation of Th1, Th2, Th17, and Treg cells in peripheral lymphoid organs (SPLs) and intestinal secondary lymphoid tissues (MLNs and PPs) of mice. LGG could promote the differentiation of Th0 cells into Th1, Th2, and Th17 cells and enhance the immunity of mice. The effect of the high-dose LGG treatment was stronger than that of the low-dose treatment. The LLGG treatment could significantly increase the proportion of Treg cells in the SPL and reduce that observed in PPs, while the HLGG treatment could significantly increase the proportion of Treg cells in PPs and reduce that observed in the MLNs, thereby regulating the balance between tolerance and immunity.

## Discussion

The animal intestinal tract is inhabited by a large number of microflora, which establish a stable and mutually beneficial symbiotic relationship between themselves and the host and maintain a dynamic balance in the intestinal tract. Previous studies have shown that *Lactobacillus* can colonize the intestinal tract, regulate the intestinal flora, increase the number of the dominant intestinal flora, improve intestinal health, and effectively prevent the infection of pathogens. Based on high-throughput sequencing analysis, LGG ATCC53103 fed to gnotobiotic pigs was shown to maintain intestinal balance and increase the dominant intestinal flora, especially to maintain the basal level of enterococci and effectively resist rotavirus infection (Zhang et al., 2014). Similarly, the results of the present study further confirmed the effect of LGG ATCC53103 on the intestinal flora in mice. After the oral administration of LGG to BALB/c mice at a high-dose (HLGG group) and low-dose (LLGG group), the observed OTU, Chao1, ACE, Simpson, and Shannon indices were obtained according to the sequencing results. Both the LLGG and HLGG treatments increased the observed OTU, Chao1, ACE, and Shannon indices and decreased the Simpson index, indicating that LGG can promote the abundance and diversity of the intestinal flora. LGG increases the number of *Firmicutes*, most of which are gram-positive beneficial bacteria, while decreasing the number of *Proteobacteria*, which are mostly gram-negative intestinal pathogens, providing intestinal probiotics a dominant community position in mice in. In addition, the results showed that the abundance and diversity of the intestinal flora in the LLGG group was higher than that observed in the HLGG group. LAB in the intestinal tract primarily regulate the balance of the intestinal flora through occupation, competition, secretion and pH value regulation. Excessive LAB will also produce conditions that are not conducive to the growth of probiotics, such as too low of a pH value, making it is necessary to further study the optimal dose of LGG to better promote the abundance and diversity of the intestinal flora.

In the present study, we showed that LGG can increase the number of CD4^+^ T lymphocytes in a dose-dependent manner, contributing to the differentiation of Th cells and enhancing cellular immune function. Th1 cells primarily secrete IFN-γ, IL-2, and TNF-α, which can enhance the cell-mediated immune response, while Th2 cells primarily secrete IL-4, IL-5, and IL-13 to enhance the humoral immune response. Th1/Th2 cells maintain a dynamic balance and can maintain normal cellular and humoral immune responses. Some studies have shown that LAB can affect the Th1/Th2 immune response (Borchers et al., 2002). When we analyzed the immune responses of mice treated with LGG, we observed that LGG could promote the expression and secretion of IFN-γ by Th1 cells and of IL-4 by Th2 cells. The ELISA results showed that LGG promoted the secretion of IFN-γ and IL-4 in serum in a dose-dependent manner, which further confirmed the flow cytometry results. The analysis of the ratio of IFN-γ/IL-4 between the LGG and control groups showed that the ratio of IFN-γ/IL-4 in the LGG group was more than 1, and there was a significant difference between the two groups (*p* < 0.05), suggesting that LGG tends to mediate the Th1 immune response. In addition, the results of the present study showed that LGG upregulated the expression of IL-17 in CD4^+^ T cells. An increase in IL-17 promotes the ability of the host to resist infection by bacteria, mycobacteria and fungi (Curtis and Way, 2009). LGG can promote the differentiation of Th0 cells into Th17 cells, promote the expression of IL-17, regulate immunity and enhance the defenses of the host. We demonstrated that LGG can also regulate the differentiation of Treg cells. Chen et al. showed that LGG can significantly improve the differentiation and promote the balance of Th-17 and Treg cells as well as alleviate alcohol-induced liver injury (Chen et al., 2016). These results indicated that LGG can not only regulate the balance between Th1 and Th2 but also regulate the balance between Th-17 and Treg. By regulating the host immune status, LGG may improve the host defense against pathogenic microbial infection, which can be evaluated by an infectious model in the future.

To sum up, compared with HLGG, LLGG can better improve the abundance and diversity of intestinal flora, but HLGG group can promote the number and function of T lymphocytes more than LLGG group. Other studies have shown that a mixture of *Lactobacillus* and *Bifidobacterium* at low concentrations (<1 × 10^6^ CFU/mL) enhances IFN-γ production and inhibits IL-4 production in mitogen activated mouse and human spleen T cells, while high concentrations (≥1 × 10^6^ CFU/mL) inhibit mitogen-induced T cell proliferation (Li et al., 2011). In this study, this phenomenon is related to the dose of LGG. We need to further study the optimal dose of LGG to maximize the abundance and diversity of intestinal flora and make the host have the best immunity. Lactic acid bacteria are increasingly used to improve human health, relieve disease symptoms, and improve the efficacy of vaccines. Our results show that the more probiotics, not always the better. It may be harmful if the optimal dose is not used for the appropriate purpose. The importance of dose selection should be emphasized in the research of probiotics in the future. The mechanism of dose effect of probiotics needs to be further studied.

## Materials and Methods

### Bacterial Strains

LGG ATCC 53103 was grown in De Man, Rogosa, and Sharpe (MRS) broth for 12 h at 37°C. After culturing overnight, the bacteria were inoculated 1:100 in fresh MRS broth and grown under anaerobic conditions until reaching the mid-log phase. Then, the colonies were counted, and the cell density was adjusted to 5 × 10^9^ colony forming units (CFU)/ml.

### Animal Experimental Procedure for LGG Intervention

Forty-five 6-week-old female BALB/c mice were randomly divided into 3 groups, with 15 mice in each group. The mice in the normal control group (Control) were intragastrically administered PBS (200 μL) every other day for 7 total treatments. The mice in the low-dose LGG group (LLGG) were intragastrically administered LGG every other day for 7 total treatments, with intervention doses of 10^3^, 10^4^, 10^5^, 10^6^, 10^7^, 10^8^, and 10^9^CFU. The mice in the high-dose LGG group (HLGG) were intragastrically administered 10^9^CFU every other day for 7 total treatments. One week after the LGG interventions (on the 21st day), the 45 from the three groups were sacrificed.

### DNA Extraction, PCR Amplification, 16S rRNA Sequencing

Total genome DNA from samples was extracted using CTAB method. DNA concentration and purity was monitored on 1% agarose gels. According to the concentration, DNA was diluted to 1 ng/μL using sterile water. 16S rRNA genes of distinct regions (16S V3-V4) were amplified used specific primer (341F: 5′-CCTAYGGGRBGCASCAG-3′ and 806R: 5′-GGACTACNNGGGTATCTAAT-3′) with the barcode. All PCR reactions were carried out with 15 μL of Phusion^®^ High-Fidelity PCR Master Mix (New England Biolabs), 2 μM of forward and reverse primers and about 10 ng template DNA. Thermal cycling consisted of initial denaturation at 98°C for 1 min, followed by 30 cycles of denaturation at 98°C for 10 s, annealing at 50°C for 30 s, elongation at 72°C for 30 s, and finally 72°C for 5 min. Mix same volume of 1X loading buffer (contained SYB green) with PCR products and operate electrophoresis on 2% agarose gel for detection. PCR products was mixed in equidensity ratios. Then, mixture PCR products was purified with Qiagen Gel Extraction Kit (Qiagen, Germany). Sequencing libraries were generated using TruSeq^®^ DNA PCR-Free Sample Preparation Kit (Illumina, United States) following manufacturer’s recommendations and index codes were added. The library quality was assessed on the Qubit@ 2.0 Fluorometer (Thermo Fisher Scientific) and Agilent Bioanalyzer 2100 system. At last, the library was sequenced on an Illumina NovaSeq platform and 250 bp paired-end reads were generated.

### Bioinformatics and Sequencing Data Analysis

After sequencing, paired-end reads was assigned to samples based on their unique barcode and truncated by cutting off the barcode and primer sequence. Paired-end reads were merged using FLASH (V1.2.7)^1^ (Magoč and Salzberg, 2011). Quality filtering on the raw tags were performed under specific filtering conditions to obtain the high-quality clean tags (Bokulich et al., 2013) according to the QIIME (V1.9.1)^2^ (Caporaso et al., 2010)quality controlled process. The tags were compared with the reference database (Silva database)^3^ using UCHIME algorithm^4^, (Edgar et al., 2011) to detect chimera sequences, and then the chimera sequences were removed (Haas et al., 2011). Then the Effective Tags finally obtained. Sequences analysis were performed by Uparse software (Uparse v7.0.1001)^5^ (Edgar, 2013). Sequences with ≥97% similarity were assigned to the same OTUs. Representative sequence for each OTU was screened for further annotation. For each representative sequence, the Silva Database (see text footnote 3) (Quast et al., 2013) was used based on Mothur algorithm to annotate taxonomic information. Alpha diversity is applied in analyzing complexity of species diversity for a sample through 4 indices, including Chao1, Shannon, Simpson, and ACE. All this indices in our samples were calculated with QIIME (Version 1.7.0) and displayed with R software (Version 2.15.3). Heatmap and network analysis were performed to visualize the taxon abundance at the genus level using graphviz software (version 2.38.0) and R software (Version 2.15.3), respectively. Linear discriminant analysis (LDA) effect size (LEfSe) was used to detect significant changes in relative abundance of microbial taxa among different groups. A significance value of less than 0.05 and an LDA effect size of greater than 3 were used as thresholds for the LEfSe analysis. Multivariate data analysis, including Venn analysis of shared and unique OTUs and partial least squares-discriminant analysis (PLS-DA), was performed using R and Simca-P 12.0 (Umetrics, Umeå, Sweden), respectively. The raw reads were deposited into the NCBI Sequence Read Archive database (accession: PRJNA 675996).

### Collection of Tissue Samples and Preparation of Single Cell Suspensions for Flow Cytometry

The spleen, mesenteric lymph node and Peyer’s patches of mice were dissected out using ophthalmic scissors and forceps and placed in a 200-mesh aseptic filter, which was placed in a sterile plate with 1 mL of RPMI-1640 culture medium. The tissue was gently and fully ground with the end of a sterile 1-mL syringe, transferred to an aseptic 1.5-mL EP tube with a pipette, and centrifuged at 4°C and 2,000 rpm for 5 min, after which the supernatant was discarded, and lymphocytes were collected. The lymphocytes were wash once with FACS buffer, resuspended in 500 μL of red blood cell lysate, and lysed at room temperature for 2 min. Then, 500 μL of RPMI-1640 culture medium was added to stop lysis, after which the samples were centrifuged at 4°C and 2,000 rpm for 5 min, and the supernatant was discarded. The lymphocytes were then collected, washed with FACS buffer twice, resuspended in 1 mL PBS and counted with a cell counting chamber.

A 10-cm section of small intestine harboring Peyer’s patches was placed in cold PBS (without Ca^2+^ and Mg^2+^). Then, the fat was removed, and the small intestine was cut longitudinally. Subsequently, the intestinal tissue was rinsed with precooled PBS (without Ca^2+^ and Mg^2+^) at 4°C until clean, after which it was transversely cut into intestinal segments 0.5∼1 cm in length. The intestinal segments were then placed in 5 mL of intraepithelial lymphocyte (IEL) separation solution (10 mM DTT, 2 mM EDTA, and 3% FBS in RPMI 1640 medium) and incubated at a constant temperature (37°C) with shaking (200 r/min) for 15 min. Subsequently, the intestinal segments were passed through a 200-mesh nylon filter and then added to 5 mL of IEL separation solution, after which the above filtration procedure was repeated after shaking at 37°C for 15 min. The remaining intestinal segments were placed in 5 mL of lamina propria (LP) digestion solution (1.5 g/L collagenase IV, 3 mg/L neutral protease (Dispase), 100 kU/L DNase I, and 5% FBS in RPMI 1640 medium). After shaking (200 r/min) at a constant temperature (37°C) for 45 min, the remaining intestinal segments were filtered with a 300-mesh aseptic filter, and the solid residue was discarded, with the filtrate collected in a 15-mL aseptic tube. After centrifugation at 4°C and 400 × *g* for 10 min, the supernatant was discarded to collect the LP cells. Then, 4 mL of an 80% isotonic Percoll solution was placed at the bottom of a 15 mL centrifuge tube, and the LP cell pellets were resuspended in 7 mL of a 40% isotonic Percoll solution. The resuspended LP cell pellets were fully mixed, pipetted onto the 80% isotonic Percoll solution, and then centrifuged at 20°C and 2,300 rpm for 20 min. Subsequently, the upper liquid layer was discarded, and the cells between two liquid levels were transferred to a new 15-mL centrifuge tube, after which PBS (without Ca^2+^ and Mg^2+^) was added to a volume of 15 mL. After centrifugation at 4°C and 2,000 rpm for 8 min, the supernatant was discarded. Then, the remaining cell pellets were washed twice with PBS (without Ca^2+^ and Mg^2+^), resuspended and counted.

### Flow Cytometry

For flow cytometry analysis, the following antibodies were used: PerCP-Cy5.5-B220 (clone: RA3-6B2), PerCP-CD3 (clone: 17A2), phycoerythrin (PE)-CD3 (clone: 17A2), fluorescein isothiocyanate (FITC)-CD4 (clone: GK1.5), allophycocyanin (APC)-CD8 (clone: 53-6.7), PE-CD25 (clone: PC61), PE-IFN-γ (clone: XMG1.2), APC-IL-4 (clone: 11B11), PE-Cy7-IL-17 (clone: TC11-18H10), and APC-Foxp3 (clone: MF23). All antibodies were purchased from BD Biosciences (San Jose, CA, United States).

For intracellular cytokine staining, 1∼2 × 10^6^ single cell suspensions were seeded into the 48-well cell culture plate, and 0.5 mL of complete medium was added to each well. Each well was incubated with 20 ng/mL of phorbol 12-myristate 13-acetate (PMA; Sigma) and 1 μg/mL ionomycin (Sigma) at 37°C for 4 h, after which GolgiStop (BD Biosciences) was added and the samples were incubated for 2 h. Then, the cells were resuspended in 100 μL PBS (without Ca^2+^ and Mg^2+^) and incubated with 10 μL of specific CD3 and CD4 monoclonal fluorescent antibodies at room temperature for 30 min away from light. Subsequently, the cells were washed twice with the proper amount of PBS (without Ca^2+^ and Mg^2+^) and centrifuged at 4°C and 2,000 rpm for 5 min, after which the supernatant was discarded. Then, the cell pellets were mixed well with 250 μL of fixation/permeabilization solution and incubated at 4°C for 20 min. One milliliter of 1 × BD Perm/Wash^TM^ buffer was directly added to each tube, and after centrifugation at 4°C and 2,000 rpm for 5 min, the cells were washed twice, and resuspended in a volume of 100 μL. Subsequently, 10 μL monoclonal fluorescent antibodies against IFN-γ, IL-4, and IL-17 was added to each sample tube and incubated at 4°C for 30 min after mixing. Each tube was then washed twice with the proper amount of PBS (without Ca^2+^ and Mg^2+^), leaving 300 μL of buffer to resuspend the cells. Subsequently, the cell suspension was passed through a 300-mesh nylon filter membrane into the flow and detected on an LSR-FORTESA flow cytometer (BD Biosciences).

For CD4^+^CD25^+^Foxp3^+^ Treg staining, 1 × 10^6^ single cell suspensions were incubated with 10 μL of specific monoclonal fluorescent antibodies against CD4 and CD25 and at room temperature without light for 30 min. Then, 1 mL of freshly prepared 1 × Fix/Perm Buffer working solution was directly added to each tube. The tubes were mixed by shaking for 3 s and then incubated at 4°C without light for 40 min. The fixed and permeabilized cells were resuspended in 1 mL of 1 × Perm/Wash Buffer, centrifuged at 4°C and 350 × *g* for 6 min, and then the supernatant was discarded. The cell pellets were resuspended in 2 mL 1 × Perm/Wash Buffer and centrifuged at 4°C and 350 × *g* for 6 min, and the supernatant was discarded. The cells were then resuspended in 100 μL of 1 × Perm/Wash Buffer, mixed with an appropriate amount of specific anti-Foxp3 antibody, vortexed for 10 s to mix well and then incubated at 4°C without light for 40 min. Each tube was then washed twice with 2 mL of 1 × Perm/Wash Buffer and centrifuged at 4°C and 350 × *g* for 6 min, and the supernatant was discarded. The cell pellets were then resuspended in 300 μL of PBS (without Ca^2+^ and Mg^2+^), passed through a 300-mesh nylon filter membrane into a tube and detected on an LSR-FORTESA flow cytometer (BD Biosciences). The data were analyzed using FlowJo 9.3 (Tree Star, Ashland, OR, United States).

### Detection of IFN-γ and IL-4 in Serum by ELISA

The IFN-γ and IL-4 levels in serum were detected using a commercial ELISA kit (R&D SYSTEMS) following the manufacturer’s instructions. Briefly, the standard was diluted and loaded according to the manufacturer’s instructions, and the blank and sample wells were appropriately prepared. On the precoated plate, 50 μL of the diluted sample solution was added to the well of the experimental sample to be tested. The sample was added to the bottom of the plate well without touching the well wall, after which the samples were gently shaken to mix well. The sealed plate was ten incubated at 37°C for 30 min. The sealing film was then carefully removed, the was liquid discarded, and each well was filled with wash buffer and incubated for 30 s and before discarding the liquid, with the process repeated 5 times. Then, 50 μL of enzyme-labeled reagent was added to each well, except for the blank well, incubated at 37°C for 30 min and then the wells were washed five times. Subsequently, 50 μL of chromogenic agents A and B were added to each well, mixed by gently by shaking and then incubated at 37°C for 15 min without light. Then, 50 μL of terminator solution was added to each well to terminate the reaction. The plate was placed into a microplate reader, the blank well was adjusted to zero, and the absorbance of each well was measured at 450 nm.

### Statistical Analysis

The data were analyzed using GraphPad Prism 5.0. Student’s t-test was used to compare two groups, and one-way ANOVA was used to analyze more than two groups. Differences were considered significant when *p* < 0.05.

## Data Availability Statement

The original contributions presented in the study are included in the article/Supplementary Material, further inquiries can be directed to the corresponding author/s. The raw reads for 16S rRNA Sequencing were deposited into the NCBI Sequence Read Archive database (accession: PRJNA 675996).

## Ethics Statement

The animal study was reviewed and approved by the Animal Ethics Committee of Jilin Agricultural University.

## Author Contributions

CS, XC, GY, and CW conceived and designed the experiments and wrote the manuscript. CS, XY, YL, MC, and RW performed the experiments. MC, YJ, DZ, JW, and HY performed high through sequencing analysis. CS, YZ, WY, HH, and LY performed the flow cytometry analysis. All authors contributed to the article and approved the submitted version.

## Conflict of Interest

The authors declare that the research was conducted in the absence of any commercial or financial relationships that could be construed as a potential conflict of interest.

## Footnotes

1 http://ccb.jhu.edu/software/FLASH/

2 http://qiime.org/scripts/split_libraries_fastq.html

3 https://www.arb-silva.de/

4 http://www.drive5.com/usearch/manual/uchime_algo.html

5 http://drive5.com/uparse/

## Supplementary Material

Supplementary Figure 1 The curves of the OTUs obtained from 15 samples. **(A)** Rarefaction curves. **(B)** Shannon curves. **(C)** Coverage curves. **(D)** OTU rank-abundance curves. Control, samples from the control group; LLGG, low-dose LGG group; HLGG, high-dose LGG group.

Supplementary Figure 2 Two-dimensional PLS-DA score plots distinguishing the gut microbiota of BALB/c mice in response to LGG pretreatment based on the relative abundance of OTUs.

Supplementary Figure 3 Heatmap showing the differentially abundant predicted KEGG pathways.

Supplementary Figure 4 Effect of LGG on the IFN-γ/IL-4 ratio in the serum of mice. **(A)** The ratio of IFN-γ/IL-4 expressed by CD4^+^ T cells in the SPL after LGG intervention. **(B)** The ratio of IFN-γ/IL-4 expressed by CD4^+^ T cells in the MLN after LGG intervention. **(C)** The ratio of IFN-γ/IL-4 in serum.
