# Supplementary figures and images for: Probiotic Lactobacillus rhamnosus GG Promotes Mouse Gut Microbiota Diversity and T Cell Differentiation
Source: Front Microbiol. 2020 Dec 17;11:607735. doi: 10.3389/fmicb.2020.607735 (PMC7773731; doi:10.3389/fmicb.2020.607735)

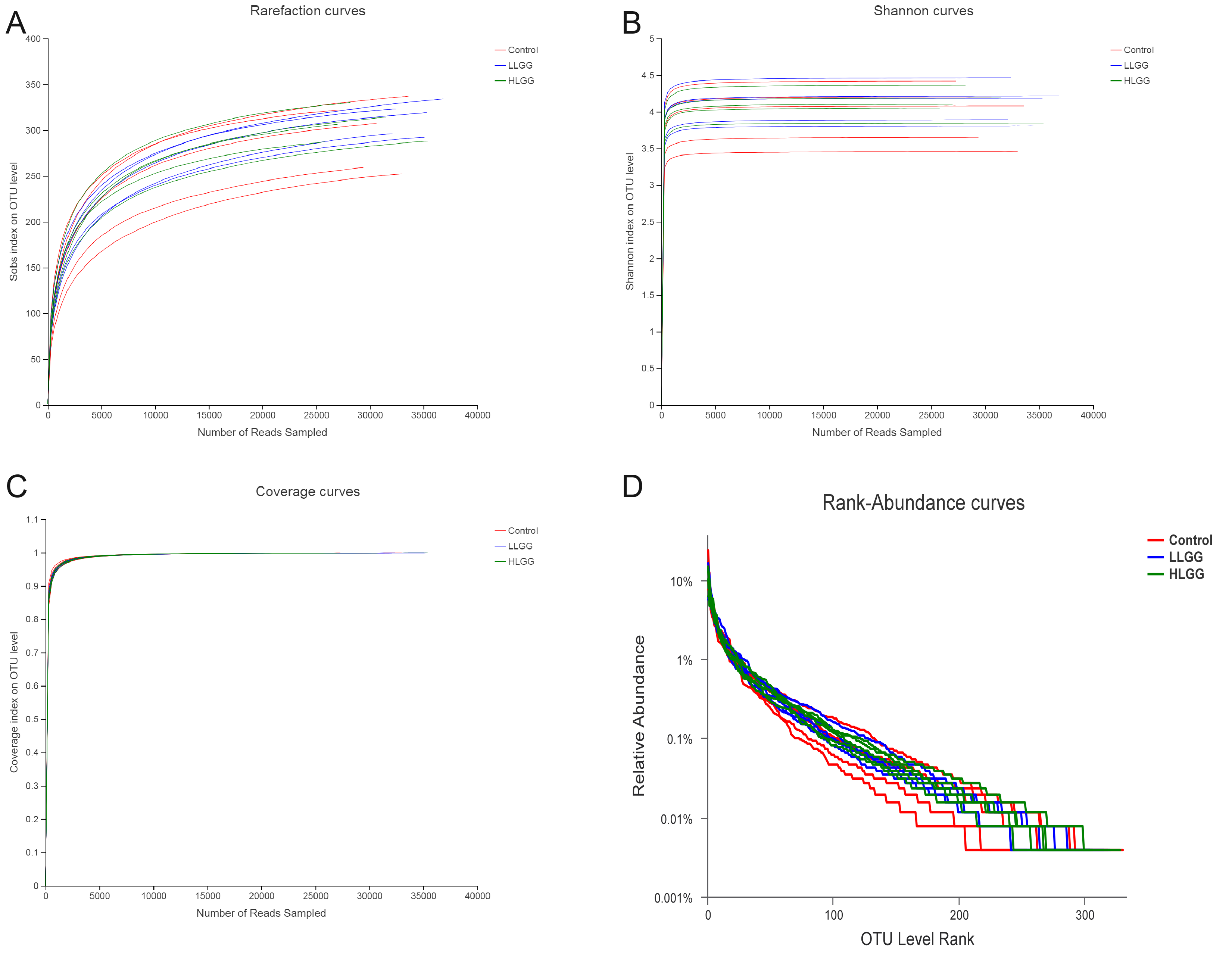

Supplement: Supplementary Figure 1 — The curves of the OTUs obtained from 15 samples. (A) Rarefaction curves. (B) Shannon curves. (C) Coverage curves. (D) OTU rank-abundance curves. Control, samples from the control group; LLGG, low-dose LGG group; HLGG, high-dose LGG group. [file Image_1.TIF]

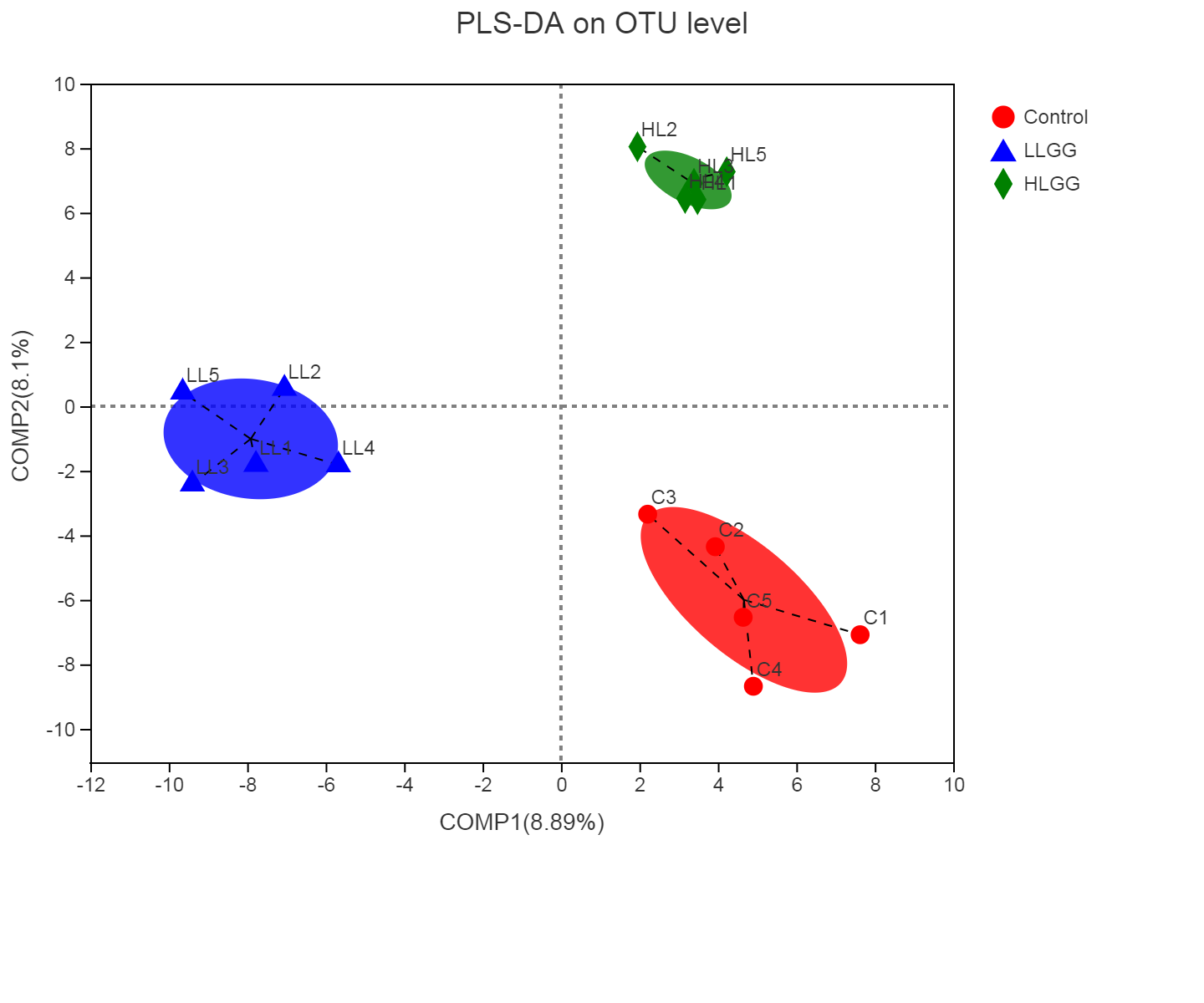

Supplement: Supplementary Figure 2 — Two-dimensional PLS-DA score plots distinguishing the gut microbiota of BALB/c mice in response to LGG pretreatment based on the relative abundance of OTUs. [file Image_2.TIF]

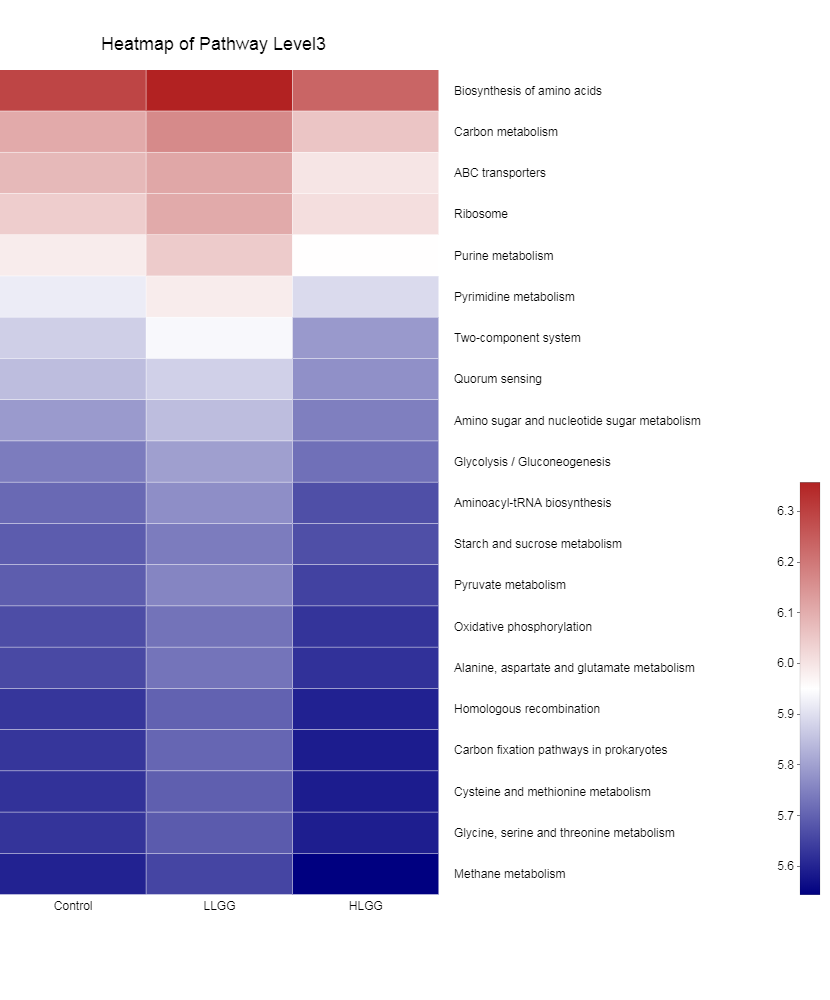

Supplement: Supplementary Figure 3 — Heatmap showing the differentially abundant predicted KEGG pathways. [file Image_3.TIF]

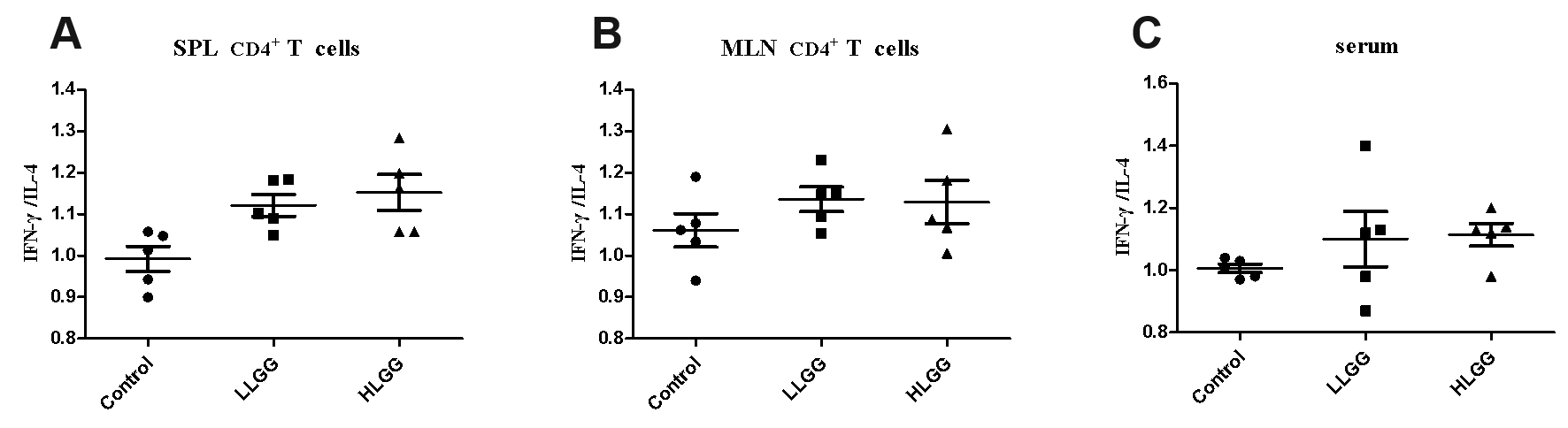

Supplement: Supplementary Figure 4 — Effect of LGG on the IFN-γ/IL-4 ratio in the serum of mice. (A) The ratio of IFN-γ/IL-4 expressed by CD4+ T cells in the SPL after LGG intervention. (B) The ratio of IFN-γ/IL-4 expressed by CD4+ T cells in the MLN after LGG intervention. (C) The ratio of IFN-γ/IL-4 in serum. [file Image_4.TIF]
